# Electric Field Induced Twisted Bilayer Graphene Infrared Plasmon Spectrum

**DOI:** 10.3390/nano11092433

**Published:** 2021-09-18

**Authors:** Jizhe Song, Zhongyuan Zhang, Naixing Feng, Jingang Wang

**Affiliations:** 1 College of Science, Liaoning Petrochemical University, Fushun 113001, China; 18641378465@163.com (J.S.); zhangzhongyuan@lnpu.edu.cn (Z.Z.); 2 Institute of Microscale Optoelectronics, Shenzhen University, Shenzhen 518060, China; 3 Key Laboratory of Electromagnetic Environmental Sensing, Department of Education of Anhui Province, Institute of Physical Science and Information Technology, Anhui University, Hefei 230601, China

**Keywords:** external electrical field, twisted bilayer graphene, surface plasmon, surface plasmon

## Abstract

In this work, we investigate the role of an external electric field in modulating the spectrum and electronic structure behavior of twisted bilayer graphene (TBG) and its physical mechanisms. Through theoretical studies, it is found that the external electric field can drive the relative positions of the conduction band and valence band to some extent. The difference of electric field strength and direction can reduce the original conduction band, and through the Fermi energy level, the band is significantly influenced by the tunable electric field and also increases the density of states of the valence band passing through the Fermi level. Under these two effects, the valence and conduction bands can alternately fold, causing drastic changes in spectrum behavior. In turn, the plasmon spectrum of TBG varies from semiconductor to metal. The dielectric function of TBG can exhibit plasmon resonance in a certain range of infrared.

## 1. Introduction

The “magic angle” graphene is similar to the structure of a heterostructure. Bilayer heterostructures have received much attention in recent years, and transition metal element heterostructures exhibit fascinating physical properties [1,2]. Similarly, twisted bilayer graphene (TBG) has also received much attention. The relative rotation angle between the double-layer graphene layers forms a twisted bilayer graphene. Cao et al. first reported the coupling and strong interaction between electrons in magic-angle graphene, which led to the observation of the quantum correlation in the entire corner double-layer graphene system [3,4,5]. In the corner double-layer graphene, from the perspective of the lattice structure, the original symmetrical rhombohedral unit cell has become a larger scale extended unit cell, and the entire system exhibits a periodic variation of Moiré superlattice (MS). In fact, the appearance of the torsion angle in the two-layer graphene determines that the Dirac cone energy bands in the two layers of graphene hybridize with each other, and the Brillouin zone of the two layers of graphene is de-twisted, and a miniature small Brillouin zone appears. In TBG, the change of the rotation angle has a decisive influence on the strong correlation and coupling of the electrons between the layers, which makes the superlattice species appear to a new quantum state, such as high temperature superconductivity, topological quantum states [6], quantum Hall effect [7], etc. The performance of surface plasmon (SP) of two-dimensional materials is comparable to that of metal surfaces, and it is more advantageous in some aspects of performance. Graphene’s potential low damping rate of plasmons [8] makes the lifespan of plasmons an obvious side length [9]. For example, in the graphene/h-BN heterostructure, the plasmon and the phonon polarization excimer are coupled to form a plasmon, and its wavelength is significantly compressed more than 100 times. There is obvious full-angle negative refraction between the mixed plasmon and the polarized excimer. The gate tunability of graphene plasmons can be combined with the high confinement property of h-BN phonon polaritons, allowing for new metamaterials that marry the unique qualities of the two materials. Additionally, the acritical demonstrated the mid-infrared (4 to 15 µm) plasmons in deeply scaled graphene nanostructures down to 50 nm, more than 100 times smaller than the on-resonance light wavelength in free space. In the superlattice of TBG, due to the strong electronic correlation of the system, the optical properties of the superlattice also have novel characteristics, such as the enhancement of fluorescence based on the corner [10], the enhancement of photochemical reaction activity [11], optoelectronics and light voltage regulation [12], optical spin Hall effect, etc. The emergence of plasmon characteristics based on the superlattice rotation angle and the study of chirality are especially more excellent [13,14,15]. Therefore, in TBG, the coupling and interaction of electrons between layers mainly depends on the distortions between the individual atomic lattices in each layer. These distortions constitute the periodic superlattice of the corner graphene, which is the Moiré superlattice in TBG. The periodicity and electrical and optoelectronic properties of TBG completely depend on the twist angle between the upper and lower layers, which makes TBG show magical and revolutionary novel characteristics in terms of electricity, optics, and heat. The TBG exhibits an abundance of high-harmonic in the intense laser fields, suggesting nonlinear “optotwistronics”, i.e., artificial twists to control the optical properties of the layered material [16]. In the TBG, some components of the nonlinear photoconductivity tensor are proportional to the orbital magnetization of the system, which would exhibit significant hysteresis behavior in the presence of a perpendicular magnetic field [17]. The twisted graphene system exhibits a large nonlinear optical response due to inversion symmetry breaking, small bandwidth, and small excitation gap. Nano-photocurrent experiments are sensitive to nanoscale changes in the Seebeck coefficient at the domain walls in minimally TBG [18]. The observed features become enhanced in a range of mid-infrared frequencies. These characteristics of the superlattice are controlled by the angle of rotation, and can also be controlled by external conditions such as electric field. Good control of material properties is a key factor for applications in nano-optical devices. By applying an electric field, the electronic properties can be effectively regulated [19,20]. The external electric field can control the energy band of TBG, thereby affecting its optical and plasmon properties. In this work, we used the first-principles methods to study the properties and physical mechanisms of plasmons and metallicity induced by an electric field outside TBG.

## 2. Methods

In this work, we use QuantumATK-2019 program package (fermitech, Beijing, China) [21] to optimize the structure of TBG and calculate the band structure, density of states, and optical properties. Among them, LCAO (linear combination of atomic orbitals) is used for the basis set, PseudoDojo is used for pseudopotentials [22], and generalized gradient approximation−Perdew Burke Ernzerhof functional (GGA-PBE) is used for functionals, combined with density functional theory with Grimme’s D3 correction without damp (DFT−D3) [23] dispersion correction. The accuracy of the computational tasks used in the text is high [24,25]. With the current calculation experience, the calculation method chosen in this paper is more accurate [26,27,28]. For the calculation accuracy, the electronic stage energy is 800 eV, and the K-point density is 2 × 2 × 1.

## 3. Results and Discussions

**Figure 1:**
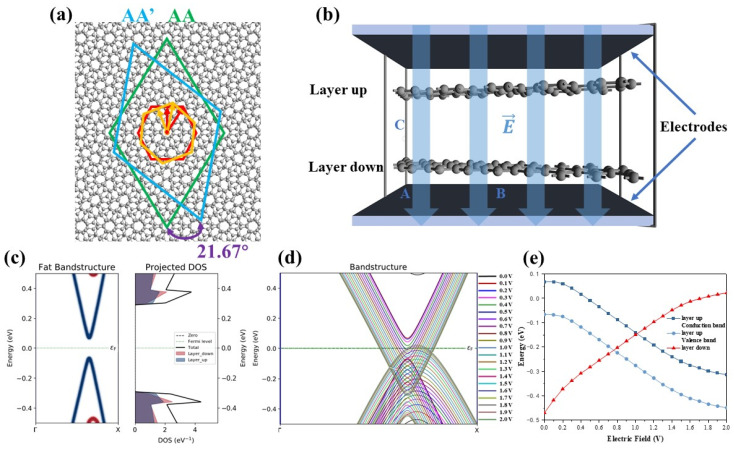
(**a**) Schematic diagram of the relative rotation of the cell and Brillouin zone of the TBG; (**b**) schematic diagram of the simulation of the optical properties of the TBG induced by external electric field; (**c**) diagram of the fat band structure and projected density of states of the TBG; (**d**) diagram of the band structures of the TBG tuned by electric field; (**e**) the band structure variations of the TBG with different electric fields.

We have theoretically investigated the optical properties of TBG under the modulation of an external electric field, with the lower layer rotated by 21.67° with respect to the upper layer (Figure 1a). We chose a particular 21.67°, the angle at which the layer-to-layer interaction forces best match. The green (AA) and blue (AA’) rhombuses represent the superlattices of the upper and lower layers, respectively. The two red and orange hexagons indicate the Brillouin zone of the upper and lower layers, respectively. The force constants obtained by the density general function theory solution combined with the pseudo-Newton method and the conjugate gradient descent method to obtain the minimum interaction distance between layers are the distance between layers. This optimization algorithm is the most general test method [29]. TBG was placed in the middle of the tunable electrode (Figure 1b). The horizontal direction in the plane is the y-direction, and the direction perpendicular to the plane is the x-direction. Before studying its optical properties and plasmon effects, we must calculate the energy bands of the structure. Figure 1c shows the energy band and the projected density of states for an electric field of 0 V. The blue and red curves in the figure are the energy band structures of the upper and lower graphene layers, respectively. The conduction and valence bands near the Fermi energy level are mainly generated by the upper graphene layer. We plotted the energy band structures in the vertical direction of the TBG under different external electric fields (Figure 1d), and the different color curves in the figure indicate the energy band structures under different electric fields. The energy bands of the upper and lower graphene layers were shifted downward and upward, respectively, under the induction of the external electric field. The electric field direction is from top to bottom as shown in Figure 1b. The difference between the energy bands of the upper and lower graphene layers modulated by the external electric field is significant. Modulated by the tunable electric field, the slope of the energy band change of the lower graphene layer is 0.25, while the slope of the conduction band and valence band change of the upper graphene layer is 0.19 (Figure 1e). The speed of the two changes with the electric field is different, indicating that the lower wave function is not sensitive to the external electric field control. This is due to the shielding effect of the ring current on the electric field caused by the aromaticity of the upper graphene π orbital. It also shows that the molar superlattice in TBG can produce greater π orbital delocalization and obtain better photoelectric performance (photoinduced charge transfer).

**Figure 2:**
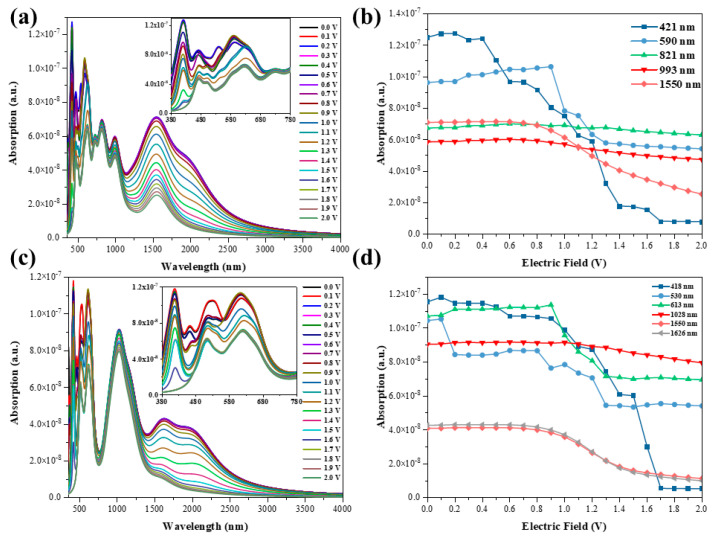
(**a**) Optical absorption spectrum of TBG induced by external electric field in xx direction; (**b**) the change of absorption intensity for tunable electric fields in xx direction; (**c**) optical absorption spectrum of TBG induced by external electric field in yy direction; (**d**) the change of absorption intensity for tunable electric fields in yy direction.

By modulating the external electric field, the energy band structure of the TBG exhibits regular changes, and we further investigated the effect of the external electric field on the optical properties (see Figure 2). The optical absorption capacity is indeed effectively modulated by the external electric field. Figure 2a,c shows the optical absorption diagrams of wavelengths from 350 nm to 4000 nm, the different color curves in the diagrams indicate the spectra obtained by different electric field modulation, and the mini diagrams are the optical absorption diagrams from 380 nm to 780 nm in the visible region. The multiple peaks of the optical absorption coefficients appear to change significantly under the external electric field induction. The peak of the absorption spectrum is due to the jump between electron orbitals, and the energy difference between the orbitals is fixed, so the position of the absorption peak does not change. Since the modulation of the energy band by the external electric field causes a change in the transition dipole moment of the electron transition, the absorption intensity changes. From Figure 2b, it can be seen that in the x orientation, the absorption coefficient decreases the most when the wavelength is 421 nm as the electric field increases, while the coefficient decreases flatly when the wavelength is 821 nm and 993 nm. At a wavelength of 590 nm, the coefficients show a flat upward trend from 0 V to 0.9 V, a large downward trend from 0.9 V to 1.3 V, and a flat trend from 1.3 V to 2.0 V. In addition, at 1550 nm, the optical absorption coefficient hardly changes at an electric field of 0 V to 0.6 V, while it shows a smooth decreasing trend at 0.6 V to 2.0 V. This is because the conduction band of the upper graphene layer keeps shifting down with the increase of the electric field, and the conduction band crosses the Fermi energy level and becomes negative when the external electric field is 0.5V (Figure 1e). It can be seen from Figure 2d that in the y orientation, the optical absorption coefficient at 418 nm is modulated by the external electric field with the greatest variation, followed by 355 nm, 530 nm, and 613 nm, while 1028 nm does not change much. In addition, at 1550 nm, the absorption coefficient does not change significantly from 0 V to 0.7 V, while it shows a decreasing trend from 0.7 V to 2.0 V. The trend at 1626 nm is close to that at 1550 nm. This is still attributed to the fact that after the external electric field is greater than 0.5V, the conduction band crosses the Fermi energy level due to the pushing effect of the external electric field on the energy band, which makes the density of states decrease and thus causes a decrease in the optical absorption coefficient.

**Figure 3:**
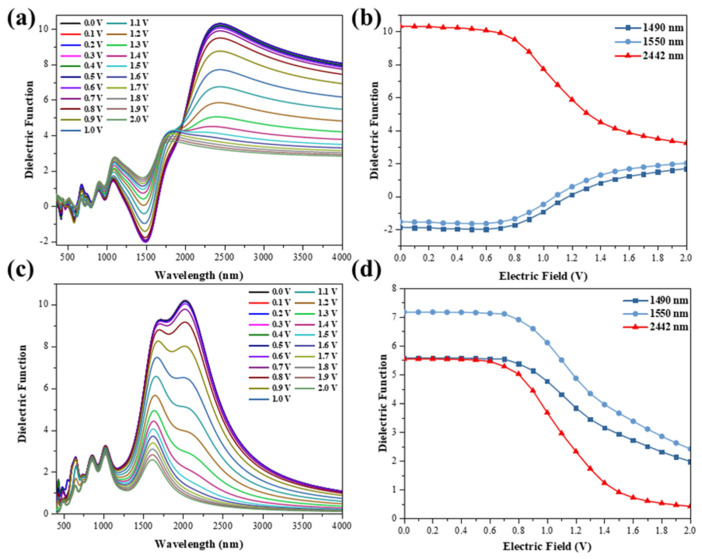
The real (**a**) and imaginary (**c**) parts of the dielectric function of the external electric field induced TBG in the x direction. The variation of the real (**b**) and imaginary (**d**) parts of the dielectric function with the tunable external electric field is analyzed. Field intensity is defined as v/nm.

**Figure 4:**
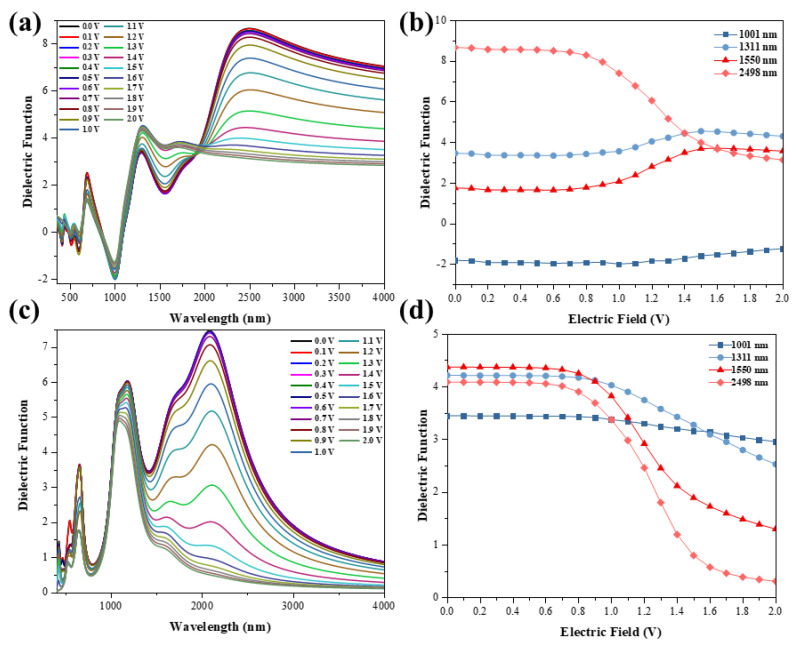
The real (**a**) and imaginary (**c**) parts of the dielectric function of the external electric field induced TBG in the y direction. The variation of the real (**b**) and imaginary (**d**) parts of the dielectric function with the tunable external electric field is analyzed. Field intensity is defined as v/nm.

**Figure 5:**
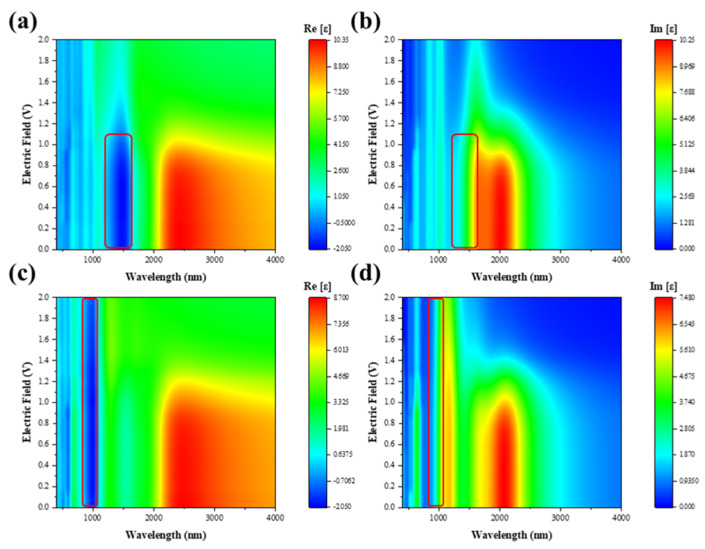
Color map of the plasmon spectrum of TBG induced by external electric field at 350−4000 nm. The (**a**,**c**) and (**b**,**d**) are the real and imaginary part of dielectric function in x and y orientation, respectively. The region in the red box will excite the surface plasmon due to the negative real part.

Usually, the optical properties of graphene regulated by an external electric field need to be applied in optical communication devices, and the dielectric function is a crucial element in the simulation process of the devices, so we calculated the dielectric function of TBG under external electric field induction (Figure 3 and Figure 4). As can be seen in Figure 3a, there is a valley in the real part of the dielectric function at 1490 nm. Additionally, the valley is significantly modulated by the external electric field. At 1550 nm, the trend of the function is similar to that of 1490 nm. Meanwhile, the peak of the real part at 2442 nm is also significantly modulated by the external electric field. In the range of 0 V to 0.6 V, for the external electric field, the real and imaginary parts of the dielectric function are almost unchanged. This phenomenon again stems from the fact that the conduction band of the upper layer has not yet crossed the Fermi energy level. While at 0.6 V to 1.5 V, the real and imaginary parts change significantly by the external electric field modulation and level off afterwards (Figure 3b,d). Meanwhile, we can see that after 1.5V in Figure 3 and Figure 4, it shows a tendency of dielectric saturation. This is because as the external electric field increases, the valence band of the lower graphene layer keeps moving upward, and when the external electric field is 1.7 V, the valence band crosses the Fermi energy level and becomes positive (Figure 1e). Moreover, when the external electric field increases to 1.0 V, the two energy bands appear to intersect due to the downward shift of the conduction band in the upper layer and the upward shift of the valence band in the lower layer (Figure 1e). This leads to a negative value (0−1 V) for the real part of the dielectric function at 1550 nm, where the TBG exhibits metallicity, and a positive value (0 V~1 V) for the imaginary part, where the structure shows a strong plasmon effect. The dielectric function in the y orientation is shown in Figure 4. With negative values in the real part and positive values in the imaginary part at 1001 nm, the TBG shows the plasmon effects. In addition, the plasmon effects and metallic regions of the external electric field-induced TBG can be seen more visually as shown in the filled color map in Figure 5. The red circle in the figure shows a strong plasmon effect and metallicity. Additionally, in the visible region, there are a few regions where the structure also exhibits metallicity and weak plasmon effects.

**Figure 6:**
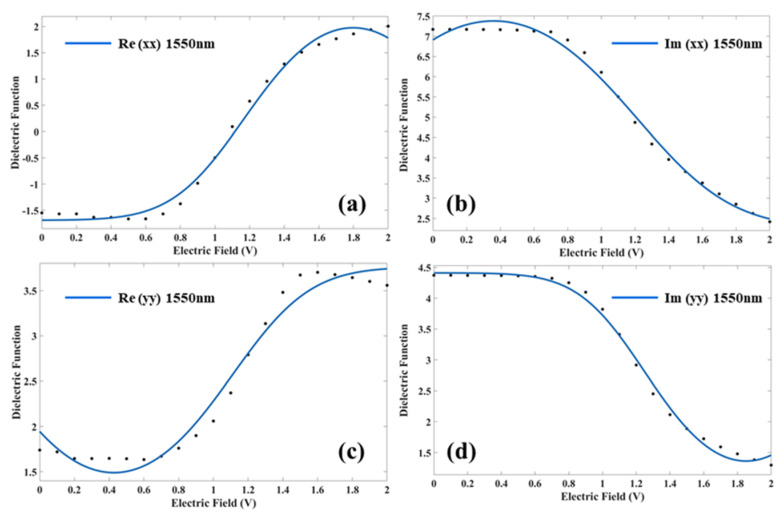
At 1550 nm, data points and fitted curves of the real portion (**a**) and imaginary portion (**b**) of the dielectric function in the x orientation under tunable external electric field, and data points and fitted curves of the real portion (**c**) and imaginary portion (**d**) of the dielectric function in the x orientation under tunable external electric field.

The mathematical laws of TBG optical properties modulated by an external electric field are of great importance for the design of modulators, waveguides, and other integrated optical circuit devices. Therefore, we use the law of quantum distribution to fit the relationship between the external electric field and the optical parameters. In fact, we have considered the effect of the Fermi distribution of temperature in the fitting process, and the fitted parameters have physical significance at this time. The fit equation of four curves using the Levenberg–Marquardt algorithm (Figure 6) is shown below:

(1) $$y=a+\frac{a_{1}}{1+e^{-a_{2}{\left(a_{3}-x\right)}^{2}/k_{B}T}}$$

where a, $a_{1}$, $a_{2}$, and $a_{3}$ are the coefficients and exponential factors, respectively; $k_{B}$ is the Boltzmann constant; *T* is the temperature; x is the value of the external electric field; and y is the value of the dielectric function at 1550 nm.

The fit coefficients and mean squared deviations (R-square) are shown in Table 1. Moreover, the standard deviation and confidence interval of the fit are also listed in the table, but R-square and the confidence interval indicate that the confidence of the current fit is very high. For large-scale simulation and device design based on current results, it will not cause large errors, and it is sufficient to qualitatively correlate the results. The trend of the fitted curve with the external electric field is consistent with the previous discussion.

## 4. Conclusions

In this work, we used the first-principles method to conduct theoretical calculations on the electronic structure and optical properties of the corner double-layer graphene (TBG) in the mid-infrared region under the control of the external electric field. First, it is found that the external electric field has the effect of reducing and increasing the conduction band and valence band of TBG, respectively. As the electric field strength increases, the energy band near the Fermi level can be adjusted in a nearly linear manner. Secondly, the external electric field can control the optical behavior of TBG by adjusting the energy band. The dielectric function near several special positions (1490 nm, 1550 nm, and 2442 nm) in the mid-infrared region is very sensitive to the regulation of the external electric field. In addition, the dielectric function near 1550 nm exhibits certain plasmon properties, which will help the design of new optical communication modulators and optical devices. We complement the scientific gap here with a fit of the parameters. In addition, the near-infrared plasmons controlled by the electric field can be used to enhance the corresponding near-infrared fluorescence and long-wavelength excited Raman spectra.

## Figures and Tables

**Table 1:** Fit coefficients of the real and imaginary parts of the dielectric functions in the x and y orientations.

	Constant Term (a)	Constant Term (a_1_)	Exponential Factor (a_2_)	Exponential Factor (a_3_)	R-Square
Re(xx)	−1.69 (−1.818, −1.562)	7.334 (6.985, 7.682)	−0.06738 (−0.08292,−0.05183)	1.798 (1.728, 1.867)	0.9924
Re(yy)	−0.7823 (−1.062, −0.5029)	4.543 (4.134, 4.952)	0.05776 (0.04048, 0.07504)	0.4275 (0.3581, 0.4969)	0.9752
Im(xx)	12.5 (12.12, 12.87)	−10.24 (−10.92, −9.555)	0.0367 (0.02884, 0.04456)	0.3609 (0.3014, 0.4205)	0.9932
Im(yy)	−1.687 (−1.884, −1.49)	6.097 (5.862, 6.333)	0.07397 (0.05935, 0.0886)	1.852 (1.791, 1.913)	0.9953

## Data Availability

The data that support the findings of this study are available from the corresponding author upon reasonable request.

## References

[1] Mu X., Sun M. (2020). Interfacial charge transfer exciton enhanced by plasmon in 2D in-plane lateral and van der Waals heterostructures. Appl. Phys. Lett..

[2] Fan J., Song J., Cheng Y., Sun M. (2021). Pressure-dependent interfacial charge transfer excitons in WSe_2_-MoSe_2_ heterostructures in near infrared region. Results Phys..

[3] Cao Y., Fatemi V., Demir A., Fang S., Tomarken S.L., Luo J.Y., Sanchez-Yamagishi J.D., Watanabe K., Taniguchi T., Kaxiras E. (2018). Correlated insulator behaviour at half-filling in magic-angle graphene superlattices. Nature.

[4] Cao Y., Fatemi V., Fang S., Watanabe K., Taniguchi T., Kaxiras E., Jarillo-Herrero P. (2018). Unconventional superconductivity in magic-angle graphene superlattices. Nature.

[5] Mele E.J. (2018). Novel electronic states seen in graphene. Nature.

[6] Choi Y., Kim H., Peng Y., Thomson A., Lewandowski C., Polski R., Zhang Y., Arora H.S., Watanabe K., Taniguchi T. (2021). Correlation-driven topological phases in magic-angle twisted bilayer graphene. Nature.

[7] Serlin M., Tschirhart C.L., Polshyn H., Zhang Y., Zhu J., Watanabe K., Taniguchi T., Balents L., Young A.F. (2019). Intrinsic quantized anomalous Hall effect in a moiré heterostructure. Science.

[8] Yan H., Low T., Zhu W., Wu Y., Freitag M., Li X., Guinea F., Avouris P., Xia F. (2013). Damping pathways of mid-infrared plasmons in graphene nanostructures. Nat. Photonics.

[9] Kumar A., Low T., Fung K.H., Avouris P., Fang N.X. (2015). Tunable Light–Matter Interaction and the Role of Hyperbolicity in Graphene–hBN System. Nano Lett..

[10] Patel H., Huang L., Kim C.-J., Park J., Graham M.W. (2019). Stacking angle-tunable photoluminescence from interlayer exciton states in twisted bilayer graphene. Nat. Commun..

[11] Liao L., Wang H., Peng H., Yin J., Koh A.L., Chen Y., Xie Q., Peng H., Liu Z. (2015). van Hove Singularity Enhanced Photochemical Reactivity of Twisted Bilayer Graphene. Nano Lett..

[12] Ta H.Q., Perello D.J., Duong D.L., Han G.H., Gorantla S., Nguyen V.L., Bachmatiuk A., Rotkin S.V., Lee Y.H., Rümmeli M.H. (2016). Stranski–Krastanov and Volmer–Weber CVD Growth Regimes to Control the Stacking Order in Bilayer Graphene. Nano Lett..

[13] Kort-Kamp W.J.M., Culchac F.J., Capaz R.B., Pinheiro F.A. (2018). Photonic spin Hall effect in bilayer graphene moiré superlattices. Phys. Rev. B.

[14] Stauber T., Kohler H. (2016). Quasi-Flat Plasmonic Bands in Twisted Bilayer Graphene. Nano Lett..

[15] Kim C.-J., Sánchez-Castillo A., Ziegler Z., Ogawa Y., Noguez C., Park J. (2016). Chiral atomically thin films. Nat. Nanotechnol..

[16] Ikeda T.N. (2020). High-order nonlinear optical response of a twisted bilayer graphene. Phys. Rev. Res..

[17] Liu J., Dai X. (2020). Anomalous Hall effect, magneto-optical properties, and nonlinear optical properties of twisted graphene systems. NPJ Comput. Mater..

[18] Sunku S.S., Halbertal D., Stauber T., Chen S., McLeod A.S., Rikhter A., Berkowitz M.E., Lo C.F.B., Gonzalez-Acevedo D.E., Hone J.C. (2021). Hyperbolic enhancement of photocurrent patterns in minimally twisted bilayer graphene. Nat. Commun..

[19] Umari P., Pasquarello A. (2002). Ab initio molecular dynamics in a finite homogeneous electric field. Phys. Rev. Lett..

[20] Wei Y., Wang F., Zhang W., Zhang X. (2019). The electric field modulation of electronic properties in a type-II phosphorene/PbI 2 van der Waals heterojunction. Phys. Chem. Chem. Phys..

[21] Smidstrup S., Markussen T., Vancraeyveld P., Wellendorff J., Schneider J., Gunst T., Verstichel B., Stradi D., Khomyakov P.A., Vej-Hansen U.G. (2019). QuantumATK: An integrated platform of electronic and atomic-scale modelling tools. J. Phys. Condens. Matter.

[22] van Setten M.J., Giantomassi M., Bousquet E., Verstraete M.J., Hamann D.R., Gonze X., Rignanese G.M. (2018). The PseudoDojo: Training and grading a 85 element optimized norm-conserving pseudopotential table. Comput. Phys. Commun..

[23] Grimme S., Antony J., Ehrlich S., Krieg H. (2010). A consistent and accurate ab initio parametrization of density functional dispersion correction (DFT-D) for the 94 elements H-Pu. J. Chem. Phys..

[24] Jones A.J., Muzzio R., Majchrzak P., Pakdel S., Curcio D., Volckaert K., Biswas D., Gobbo J., Singh S., Robinson J.T. (2020). Observation of electrically tunable van Hove singularities in twisted bilayer graphene from NanoARPES. Adv. Mater..

[25] Mu X., Sun M. (2020). The linear and non-linear optical absorption and asymmetrical electromagnetic interaction in chiral twisted bilayer graphene with hybrid edges. Mater. Today Phys..

[26] Dev P., Agrawal S., English N.J. (2013). Functional Assessment for Predicting Charge-Transfer Excitations of Dyes in Complexed State: A Study of Triphenylamine–Donor Dyes on Titania for Dye-Sensitized Solar Cells. J. Phys. Chem. A.

[27] Städele M., Moukara M., Majewski J.A., Vogl P., Görling A. (1999). Exact exchange Kohn-Sham formalism applied to semiconductors. Phys. Rev. B.

[28] Grimme S., Hansen A., Brandenburg J.G., Bannwarth C. (2016). Dispersion-corrected mean-field electronic structure methods. Chem. Rev..

[29] Kohn W., Sham L.J. (1965). Self-consistent equations including exchange and correlation effects. Phys. Rev..

